# Supplementary material for: Deprivation of Sexual Reproduction during Garlic Domestication and Crop Evolution
Source: Int J Mol Sci. 2023 Nov 26;24(23):16777. doi: 10.3390/ijms242316777 (PMC10706073; doi:10.3390/ijms242316777)
Supplement: Supplementary file 1 [file ijms-24-16777-s001.zip › Table S2 Origin of genotypes used in the genome analysis.pdf]

Table S2 Origin of 41 genotypes used in the genome analysis. The list is divided into four phenotypic groups, according to reproductive traits. In **bold**: eight genotypes that were used for transcriptome analysis.

Genotype	Origin	Genotype	Origin
**BOLTING AND FLOWERING GENOTYPES**			
**X-87**	**Seed-producing breeding line**	**3255**	**Czech Republic**
2000	Kazakhstan	3257	Europe
3275	Uzbekistan	**3291**	**Czech Republic**
3118	Kazakhstan	3028	Kazakhstan
3214	Central Asia	3212	Central Asia
3215	Central Asia	**3259**	**Central Asia**
3274	Russia	3273	Russia
**BOLTING GENOTYPES, INFLORESCENCE WITH TOPSETS, FLOWERS ARE ABORTED**			
**3207**	**China**	3250	Ukraine
**3242**	**China**	3249	Japan
**3243**	**China**	**3296**	**Georgia**
**SEMI-BOLTING GENOTYPES**			
2578	Kazakhstan	2284	Unknown
3299	USA/Palestinian	**3261**	**Israel - cv. Shani**
2216	China		
**NON-BOLTING GENOTYPES**			
2163	Unknown	**3241**	**USA**
2270	Italy	3231	Bulgaria
2297	Europe	3251	France
3230	Unknown	3256	Greece
3252	France	3258	Greece
3267	Bulgaria	3290	France
3298	USA/Syria	3368	Nepal
2128	France	3371	Italy
